# Active prosthesis dependent functional cortical reorganization following stroke

**DOI:** 10.1038/s41598-017-09325-8

**Published:** 2017-08-17

**Authors:** Christian Merkel, Janet Hausmann, Jens-Max Hopf, Hans-Jochen Heinze, Lars Buentjen, Mircea Ariel Schoenfeld

**Affiliations:** 1 Department of Neurology, Otto-von-Guericke University, Magdeburg, Germany; 2 Department of Stereotactic Neurosurgery, Otto-von-Guericke University, Magdeburg, Germany; 3 Department of Behavioral Neurology, Leibniz-Institute for Neurobiology, Magdeburg, Germany; 4 Kliniken Schmieder Heidelberg, Heidelberg, Germany

## Abstract

The present study investigated the neural correlates associated with gait improvements triggered by an active prosthesis in patients with drop-foot following stroke during the chronic stage. Eleven patients took part in the study. MEG recordings in conjunction with somatosensory stimulation of the left and right hand as well as gait analyses were performed shortly before or after prosthesis implantation surgery and 3–4 months later. Plastic changes of the sensorimotor cortex of the ipsi- and contralesional hemisphere were revealed. Gait analysis indicated that all patients improved their gait with the active prosthesis. Patients with larger plastic changes within the lesioned hemisphere maintained their improved gait performance even when the prosthesis was turned off. Patients with larger contralesional changes also improved their gait with the active prosthesis. However, their gait measures decreased when the prosthesis was turned off. The current data provide the neural basis of gait improvement triggered by an active prosthesis and has important implications with respect to the choice of the type of active prosthesis (implantable vs removable) and to the selection procedure of the patients (length of testing period).

## Introduction

About 10–20% of all surviving stroke patients suffer from drop-foot symptoms^1^. This largely common characteristic in hemiplegic patients displays itself in a failure to dorsiflex the foot during the swing phase of the gait^2^ and constitutes a considerable decline in quality of life.

The most common strategy to treat those patients is to fixate their foot at a 90° angle using an ankle foot orthesis (AFO)^3^. The improvement in combination with the low costs result in the widespread use of AFOs as treatment of choice for drop-foot. But even though AFOs have been shown to improve gait-parameters up to about 6 months after stroke, data also indicate that in the long run (>12 months) AFOs do not have any additional therapeutic effect on gait speed or stance balance anymore^4–6^.

Studies showed very early that functional electrical stimulation (FES) of the impaired muscle could be used as an alternative to treat drop foot^7^. Using FES, lower limb motor functionality can be improved in a variety of upper motor neuron lesions that leave the excitability of the impaired muscle fibers intact^8–10^. In the last 55 years a variety of stimulation devices have been introduced in which the main principle of peroneal excitation remained unchanged^11^. It has been described that the use of FESs results not just in an increase of actual dorsiflexion of the tibialis anterior to lift the foot^12^ but also innervates previously silent muscle fibers^13^, reduces spasticity of the affected leg^1, 14^, improves the symmetry of general gait posture^5^ and leads to a general increase in walking speed^10, 15^.

Interestingly, already the first observations of FES in drop-foot describe not only an imminent improvement of gait during stimulation, but also a ‘restoration’ of voluntary dorsiflexion in between stimulation protocols^7^. This purely therapeutic^8^ effect of FES becomes apparent especially in the long run during stimulator training^16–18^. Some studies indicate that the therapeutic effect of FES hereby seems to outperform that of AFO-training^4, 5, 19, 20^.

Authors interpreted the therapeutic gain of long-term FES use as an indication of central neural plasticity processes triggered by the stimulation^16, 17, 21^. The only data supporting this claim report a general increase of corticospinal excitability from transcranial magnetic stimulation over the motorcortex following training phases of peripheral stimulation^16, 22^.

However up to now no direct evidence for neural plasticity changes in sensorimotor cortical areas following functional electric stimulation in drop foot is available. The current study presents first electrophysiological data, directly tracing cortical reorganization processes over time, in relation with changes of gait kinematics in patients with upper motor neuron lesions in their chronic state.

## Methods

### Subjects

**Figure 1:**
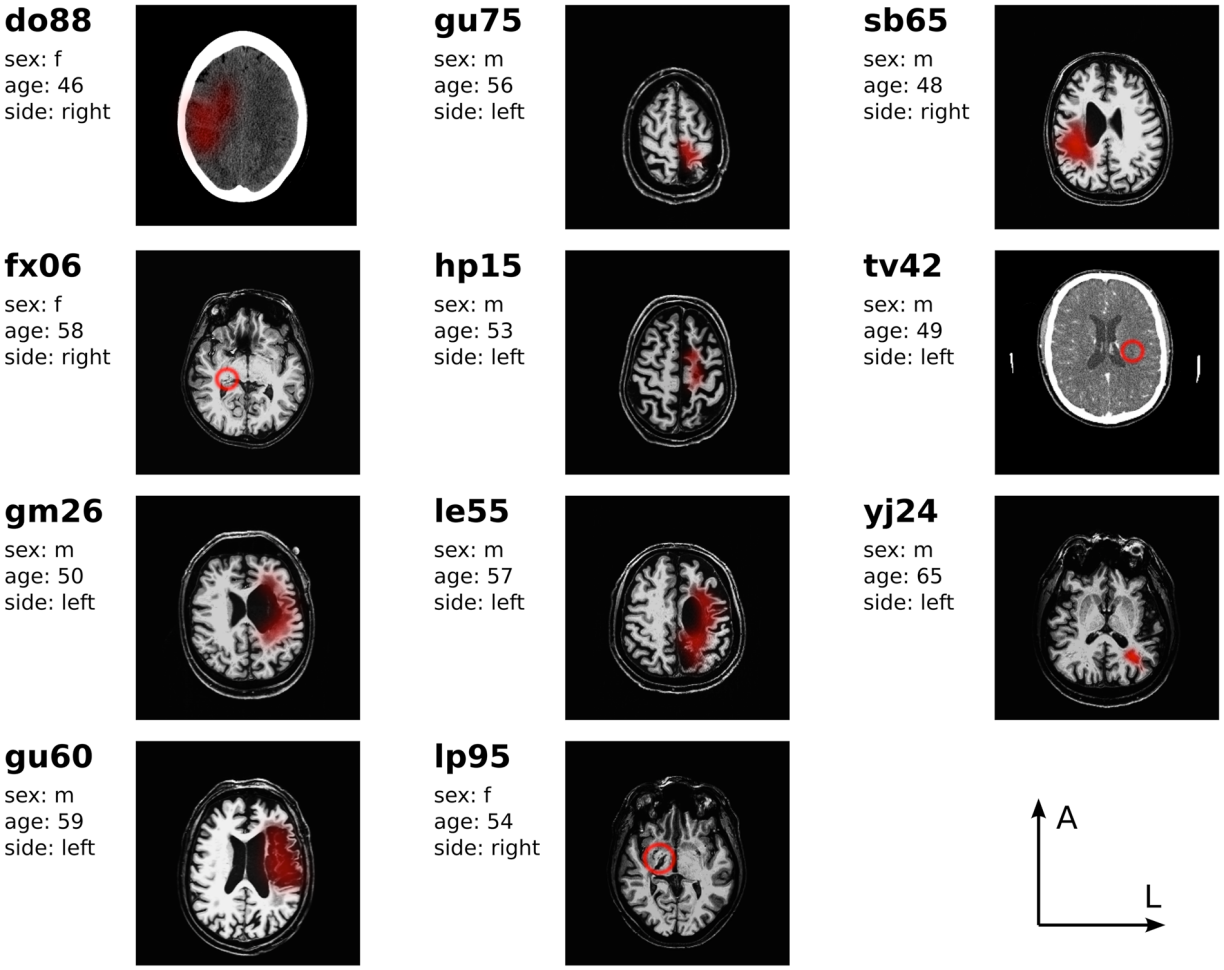
Description of eleven included patients. (do88) right hemispheric medial cerebral artery infarct including medial parts of S1 and M1 extending into the parietal lobe (fx06) right subcortical lesion touching the posterior limb of the internal capsule (gm26) posthemorrhagic lesion on the left including cortical and subcortical S1 and M1 (gu60) left-sided lesion in A.-lenticulostriata territory touching cortical and subcortical parts of prefrontal and medial S1 and M1 extending into parietal lobe (gu75) lesion following intracerebral bleeding on the left touches medial S1 and M1 extending into superior parietal cortex (hp15) infarct of the left medial cerebral artery touching the medial S1 and M1 (le55) left-sided hemorrhage touching cortical and subcortical parts of frontal, medial S1 and M1 including superior parietal cortex (lp95) ischemia of the A.-thalamostriata territory including the posterior limb of the right internal capsule (sb65) right posthemorrhagic lesion including medial S1 and M1, posterior limb of the internal capsule extending into the temporal lobe (tv42) left-sided infarct including the posterior external capsule (yj24) ischemia including the left posterior limb of the internal capsule.

Eighteen patients with upper motor lesions gave written informed consent to participate in this study that was approved by ethics committee of the medical faculty of the Otto-von-Guericke university and was carried out in accordance with the relevant guidelines and regulations. Patients were implanted with a partially implantable ActiGait system (OttoBock, Berlin). At the time of surgery all patients were in their chronic state in that all participants experienced their lesion incident more than two years ago except for one (1.7 years). Figure 1 summarizes all patients included in the following analyses together with individual imaging slices illustrating each patients’ lesion.

### Clinical assessment

Magnetencephalographic measurements as well as gait kinematic assessments were performed twice (T1/T2) with an average interval of about three to four months with T1 occurring shortly before or after operation. At both time points additional quality of life assessments with the SF-36 questionnaire were performed.

### Gait Analysis

Gait kinematics were assessed using motion capture techniques (Vicon) in combination with Vicon Nexus’ build-in Plug-In Gait algorithm based on the Newington-Helen Hayes gait model^23^. Full body joint kinematics were calculated at the two examination time points (T1 and T2) for each subject. The full body model for each subject was created to each gait-analysis using a static trial in which rigid body segment positions were automatically identified based on 34 reflective markers attached to the body at standardized positions. Subsequently, multiple dynamic gait kinematics based on the Plug-In Gait full body model were recorded in which subjects had to walk across the distance of the capture area of 10 m. Walks were recorded at T1 (shortly before or after stimulator implantation) under the condition of absent stimulation assisted gait (T1_OFF_). For T2 different walks were recorded separately for the two different gait-conditions with and without active stimulation (T2_OFF_, T2_ON_).

Each walking condition was recorded twice. Marker trajectories for each dynamic gait recording were reconstructed offline and labeled based on the previously created static gait model. Subsequently, mislabeled markers were corrected manually and gaps were filled using Vicon Nexus software. Within each preprocessed dynamic gait recording each strike and foot-off gait-cycle event was identified manually. Gait-parameters for each gait-condition were then estimated using the dynamic gait model. For each subject at each time point and gait-condition (T1_OFF_, T2_OFF_, T2_ON_) the following three gait-parameters in reference to Perry^2^ were estimated: cadence (m/s), steptime (s) and stridetime(s).

For two subjects dynamic gait recordings failed at one time point due to technical difficulties. Thus data from those subjects could not be used for further analysis.

### Structural MRI

A structural MRI was recorded for each subject prior to the implantation of the device. Due to the institutes’ safety guidelines structural MRIs could not be performed for two patients. In these patients lesions have been assessed using CT-scans. For all other patients a high resolution MPRAGE structural image was recorded using a 3 T Siemens VERIO scanner (1.0 × 1.0 × 1.0 mm, TR = 2500 ms, TE = 4.82, TI = 1100 ms, FlipAngle = 7°, FOV = 256 mm).

Structural MRIs were used to create high-resolution surface reconstructions^24^ using FreeSurfer (https://surfer.nmr.mgh.harvard.edu). Images with substantial cortical lesions were segmented using a semi-automatic white matter segmentation step *(fast* provided by FSL-library (http://www.fmrib.ox.ac.uk/fsl/)) after an initial reconstruction step to subsequently refine the final surface reconstruction.

The Surfaces were used to create individual realistic one-shell boundary element models (BEMs) and source models required for the estimation of individual distributed source models for the individual sensorimotor MEG-field distributions of each patient for each measurement.

### MEG-paradigm and recording

We sought to track changes in the cortical organization of the sensorimotor cortex (SMC) over time in ischemic patients with implanted active peroneus stimulator within the lesioned as well as the unaffected hemisphere. Therefore, we localized the cortical responses to sensory stimulation to the left and right hand and took differences between these locations across the two time points within each hemisphere as an indicator for plasticity within the SMC. Cortical motor areas are highly interlinked with the corresponding topographical sensory areas due to motor-related proprioceptive feedback loops^25^. Motor tasks for a specific limb part produce virtually the same activation as somatosensory stimulation of that same limb part in adjacent walls of the central sulcus^26, 27^.

Evoked somatosensory magnetic fields were recorded at the two assessment time points in response to a short pneumatic stimulation of either the left or right hand (delivered by a custom made pneumatic stimulator). Each hand was stimulated in a separate experimental block. Before each block the pneumostimulator-tube was attached to the corresponding hand. Stimulation for each hand was repeated 400 times with a 2000 ms inter-trial interval. Continuous magnetencephalographic data was recorded with a sampling rate of 2034.51 Hz and an online low-pass filter of 400 Hz over 248 magnetometer sites using a BTI Magnes 2500 WH (4-D Neuroimaging, San Diego, TX) whole head system. The position of all sensors relative to the head was determined at the beginning of each block by localizing the relative position of five coils attached to the head to the position of three fiducial points (nasion, LPA, RPA) digitized with a Polhemus tracker. Two patients were not able to finish one and two of the presented recording blocks at one of the examination time points, respectively, due to exhaustion. Consequently, incomplete data from those two subjects had to be excluded from further analysis.

Data preprocessing was performed with custom software using MATLAB (Mathworks) in combination with the *fieldtrip*-toolbox^28^. Offline data were epoched from −300 ms to 600 ms relative to the onset of the stimulation and subsequently baseline corrected. MEG-data of all patients contained a large amount of internal (alpha-band, heartbeat) as well as external (motion) noise. We therefore applied for each assessment time point an independent component analysis on the original, unfiltered and epoched data (across both condition blocks) to identify and separate sources of substantial noise from somatosensory evoked responses based on the components’ topographical distributions as well as their averaged evoked responses. Identification of all extracted components for all patients at both time points was performed by an expert in the field. On average 19.44 (range: 6−29) and 20.33 (range: 11–29) components were removed at the two measurement time points respectively. The number of rejected components did not differ significantly between the measurements of each subject (F(1, 10) = 0.178, p > 0.5). The reconstructed datasets were averaged over evoked signals for each block (left/right hand) and low-pass filtered at 35 Hz.

The decomposed MEG-signal of three patients at one or both time-points did not reveal clear somatosensory components, thus did not allow for a reconstruction of reliable somatosensory evoked fields. The data for these three patients were excluded from further analysis.

### Source localization

The first clear component of the evoked somatosensory responses for both hemispheres and measurement time points were modeled using Curry 7.0 Software (Neuroscan Inc.). We applied two different source reconstruction techniques in order to characterize the changes of the somatosensory hand-representations over time.

To compare the topographical changes we first modeled somatosensory field sources with a single dipole. For each individual subject evoked fields of each block and time-point were reoriented to a common spherical head-model using relative sensor locations as measured prior to each block. For each of the four field distributions (2 blocks (left/right hand) × 2 time points) the dipole position within the common spherical head-model was determined. Plasticity changes over time were then quantified as the difference vector between dipoles of the two time points within each hemisphere: ${R}_{DIST}=\,|R\_DI{P}_{T1}-R\_DI{P}_{T2}|$ and ${L}_{DIST}=\,|L\_DI{P}_{T1}-L\_DI{P}_{T2}|$. Hereby, $R\_DI{P}_{T1},R\_DI{P}_{T2},L\_DI{P}_{T1},L\_DI{P}_{T2}$ describe the dipole locations for the left and right hemisphere at the first and second MEG-session. Subsequently we compared the length of the difference vectors between the *lesioned* (*DIST*_*les*_) and *non-lesioned* (*DIST*_*noles*_) hemisphere of each subject and calculated the relative distance change across the hemispheres as: $ECD\_Index=(\frac{DIS{T}_{les}-DIS{T}_{noles}}{DIS{T}_{les}+DIS{T}_{noles}})$. This approach is similar to the standard method of quantifying hemispheric recruitment in stroke patients by measuring relative strength differences between sensorimotoric cortices (laterality index: refs 29–32).

As a second approach we estimated the distributed source solution (L2 minimum norm) for each of the somatosensory evoked fields using high-resolution surface reconstructions based on realistic source space models derived from the MRI for each individual subject. Two subjects were omitted from this analysis because high resolution MRI data could not be obtained. The evoked fields were reoriented by realigning the fiducial points measured prior to each MEG-block (preauricular points and nasion) with those identified within the corresponding MPRAGE-image. The maximum current source densities of each patient for each stimulation condition and time point were identified within the corresponding postcentral gyrus. This analysis strategy was used to illustrate plastic changes within the individual left and right somatosensory cortex of each subject.

### Statistical analyses

Changes in quality of life were assessed by comparing SF-36 scores across time points using paired t-tests for the different sub-scales. Changes of gait parameters over gait conditions (T1_OFF_, T2_OFF_, T2_ON_) were tested using repeated measures ANOVAe. To determine whether relative changes in gait-parameters over time $(\frac{T{2}_{OFF}-T{1}_{OFF}}{T{2}_{OFF}+T{1}_{OFF}})$ can be explained by basic patient characteristics, they were correlated with the patients’ descriptive variables (age, sex, lesioned hemisphere, time since incident, time between assessment time points and time between implantation and T1). The same analysis was also performed on the ECD-index, in order to determine whether cortical reorganization over time can be predicted by patients’ characteristics at time point T1.

To investigate whether successful active stimulation by the prosthesis is directly related to cortical reorganization in sensorimotor areas, the dipole-Index was correlated with the relative changes of gait parameters over time $(\frac{T{2}_{OFF}-T{1}_{OFF}}{T{2}_{OFF}+T{1}_{OFF}})$ and across stimulation condition at T2 $(\frac{T{2}_{ON}-T{1}_{OFF}}{T{2}_{ON}+T{1}_{OFF}})$. Furthermore, patients were divided into two subgroups of subjects that showed higher cortical reorganization within the lesioned hemisphere (ECD-Index > 0, N = 6) and non-lesioned hemisphere (ECD-Index < 0, N = 5). Subsequently changes in gait-parameters between those two groups were examined over time and over stimulation condition at T2, using 2-factorial mixed repeated measures ANOVAs (Group x time point (T1_OFF_, T2_OFF_); Group × stim (T2_OFF_, T2_ON_)). F-values from any rANOVAs introducing the three-level factor gait-condition were Greenhouse-Geisser corrected.

## Results

### Descriptive Statistics

Patients’ age ranged from 46.1 to 64.7 years with $\mathop{x}\limits^{\bar{}}$ = 54.1 and $\sigma$ = 5.5 years at the time of device implantation. The lesion occurred on average 5.8 ($\sigma$ = 3.1) years before implantation, ranging from 1.7 to 12.0 years. Two patients’ lesion occurred 2 and 3.8 years before implantation, respectively. Four subjects suffered from a cerebral damage within the right hemisphere while in 7 subjects the lesion was located within the left hemisphere. The first measurements (T1) for most patients were performed from two days prior to 5 days after the implantation. In three patients the first examination time point (T1) occurred 16, 38 and 177 days after operation, respectively. In nine subjects the second examination (T2) occurred within 82 to 139 days after T1. For two subjects the time between T1 and T2 amount to 191 and 366 days respectively.

### Inferential Statistics

**Figure 2:**
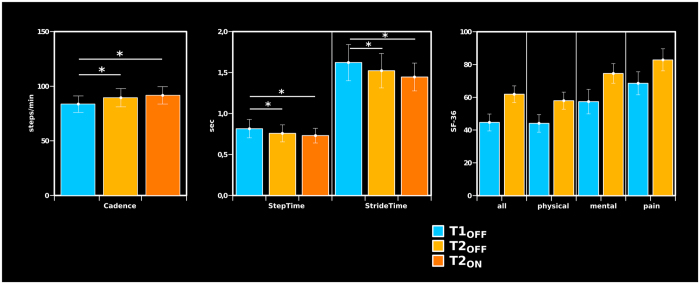
Behavioral results. Gait parameters and SF-36 questionnaire across assessment time point and stimulation condition. Error bars depict the standard error of mean.

Results from the SF-36 subscores where highly consistent. A repeated measures ANOVA for the relative changes over time across the subscores did not reveal any significant differences in the change over time between the subscores (F(3, 30) = 0.94, p > 0.4), suggesting a high inter-item reliability. Quality of life improved significantly for patients from T1 to T2 as shown by the changes of SF36-subscores (all: t(10) = 4.06, p < 0.01; physical: t(10) = 3.04, p < 0.02; emotional: t(10) = 2.89, p < 0.02; pain: t(10) = 2.40, p < 0.05) (Fig. 2).

Out of the gait-parameters (T1_OFF_, T2_OFF_, T2_ON_) the cadence showed significant differences across gait conditions (F(2, 20) = 8.75, p < 0.01). Post-hoc paired t-tests showed a significant improvement of the cadence from T1_OFF_ to T2_OFF_ (t(10) = 2.75, p < 0.02) and from T1_OFF_ to T2_ON_ (t(10) = 3.27, p < 0.01). The increase in cadence from T2_OFF_ to T2_ON_ failed to reach significance (t(10) = 1.93, p = 0.082). Likewise steptime and stridetime values differed across the three gait conditions (steptime: F(2, 20) = 8.05, p < 0.012; stridetime: F(2, 20) = 6.36, p < 0.025) (Fig. 2). Steptime and stridetime improved from T1_OFF_ to T2_OFF_ (steptime: t(10) = −3.70, p < 0.01; stridetime: t(10) = −3.55, p < 0.01) and from T1_OFF_ to T2_ON_ (steptime: t(10) = −2.91, p < 0.02; stridetime: t(10) = −2.69, p < 0.03). However those two parameters as well failed to change significantly from T2_OFF_ to T2_ON_ (steptime: t(10) = −1.55, p > 0.15; stridetime: t(10) = −1.61, p > 0.13).

Subsequently the relative change of those gait-parameters over time (T1_OFF_ vs. T2_OFF_) was correlated with the individual descriptive variables. None of the parameters (cadence, steptime and stridetime) changed over time for different sex (r(11) = −0.13, p > 0.7; r(11) = 0.09, p > 0.7; r(11) = 0.12, p > 0.7 respectively), side of the lesion (r(11) = −0.41, p > 0.2; r(11) = 0.36, p > 0.2; r(11) = 0.37, p > 0.2) or age (r(11) = −0.08, p > 0.8; r(11) = 0.11, p > 0.7; r(11) = 0.06, p > 0.8). Most importantly the change in gait-parameters over time cannot be explained by the amount of years since the lesion occurred (r(11) = 0.36, p > 0.3; r(11) = −0.21, p > 0.5; r(11) = −0.33, p > 0.3), the time between implantation and first measurement (r(11) = 0.23, p > 0.5; r(11) = −0.16, p > 0.6; r(11) = −0.20, p > 0.5) or the duration between first and second assessment time point (r(11) = 0.18, p > 0.5; r(11) = −0.22, p > 0.5; r(11) = −0.19, p > 0.5). The same holds true for the relative change in gait parameters over stimulation (T2_OFF_ vs. T2_ON_). The amount to which patients gait improved with active stimulation at T2 was also independent of the years elapsed since the lesion had occurred (r(11) = −0.12, p > 0.7; r(11) = 0.02, p > 0.9; r(11) = 0.08, p > 0.8) and the time between assessment time points (r(11) = −0.19, p > 0.5; r(11) = 0.47, p > 0.1; r(11) = 0.24, p > 0.4). Additionally, the initial gait performance at T1 was unrelated to the duration between the implantation and T1 (cadence: r(11) = 0.11, p > 0.75; steptime: r(11) = −0.17, p > 0.6; stridetime: r(11) = −0.16, p > 0.6).

**Figure 3:**
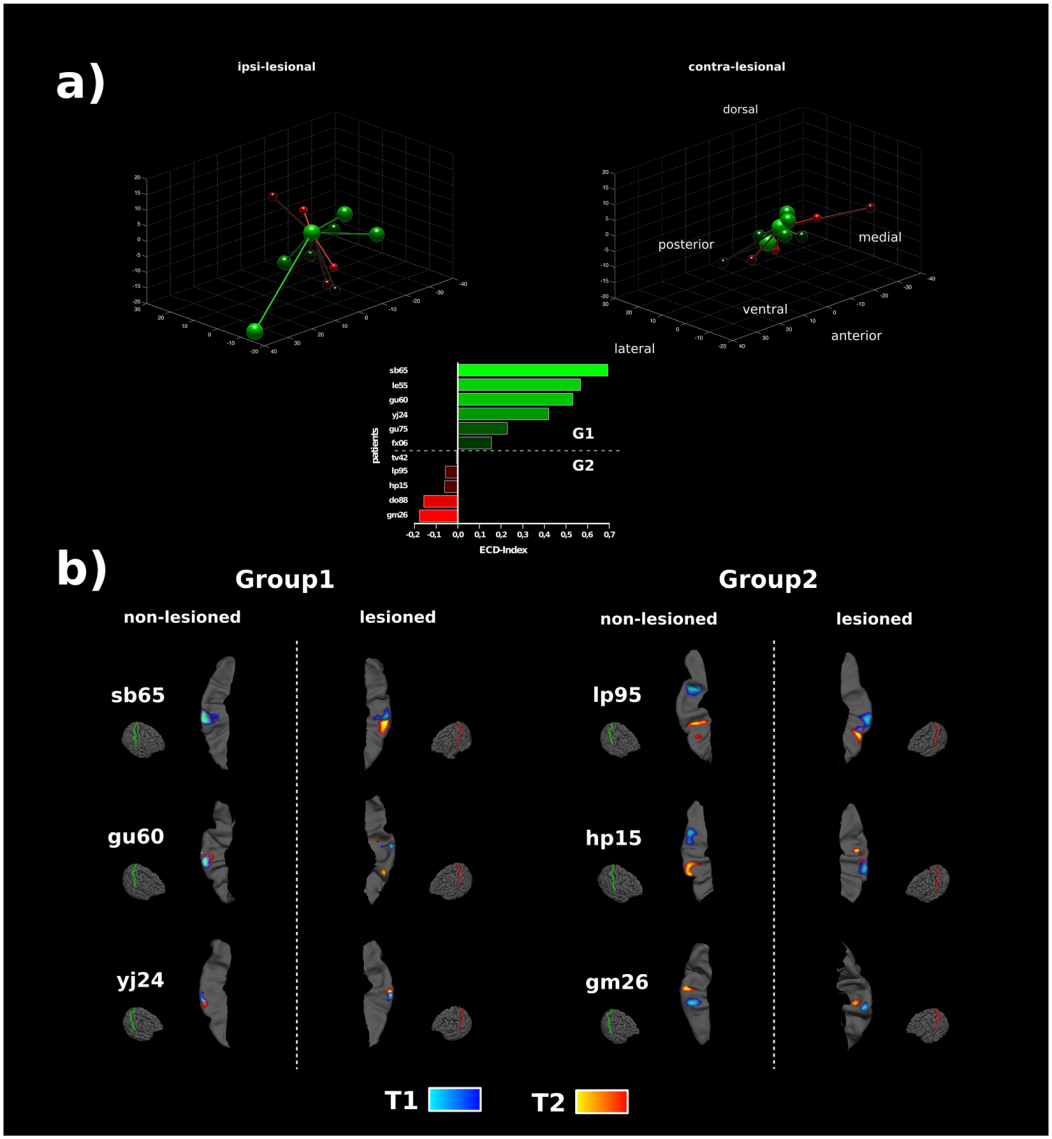
Source localizations. Ipsi- and contralesional source displacements over time (**a**) based on the equivalent single current dipole model. Absolute displacements of sources of all patients within a common space. [0, 0, 0] at both sides denotes the dipole position at the time of the first MEG measurement (T1) in each patient. Larger relative displacements over time for the ipsilesional hemisphere are colored in green shades while larger contralesional displacements are colored in red shades. The bar plot below illustrates the relative dipole displacements over time for each single patient. The ECD-Indices show more negative values for larger displacements contra-lesional and more positive values for larger displacements within the ipsilesional hemisphere. (**b**) Results of the individual distributed source modeling on realistic cortex models. The maximum of the source estimates for T1 and T2 is shown on the ipsi- and contralesional postcentral gyrus. Hemispheres are flipped in that the left side illustrates the lesioned hemisphere in all patients.

Using MEG source localization, cortical reorganization from T1 to T2 was quantified as relative distance change of dipoles within the lesioned and non-lesioned hemispheres $ECD\_Index=(\frac{DIS{T}_{les}-DIS{T}_{noles}}{DIS{T}_{les}+DIS{T}_{noles}})$. Six patients showed a positive index, thus a larger displacement of the sensory hand area within the lesioned hemisphere across time (Fig. 3a). In most of those patients this displacement occurred towards ventral according to the absolute dipole positions at T1 and T2 (Fig. 3a,b). The qualitative source distributions for those subjects largely overlap with this observation, as the ipsi-lesional sources mainly show a ventral shift (Fig. 3b). A larger shift of the contra-lesional hand area during the same time period (negative ECD-index) was observed in five patients. The direction of the shift appears to be more heterogeneous in this group (Fig. 3a,b). First, the ECD_Index was correlated with the patient’s descriptive variables. Similar to the previous correlation analysis regarding changes of gait parameters over time, the relative cortical changes over time were analyzed with respect to the same patients’ characteristics. The plasticity index showed no correlation with gender (r(11) = 0.43, p > 0.15), side of lesion (r(11) = 0.09, p > 0.7) or age (r(11) = 0.43, p > 0.15). Similarly, the amount of time between assessment time points (r(11) = 0.24, p > 0.45), the time since the lesion occurred (r(11) = 0.42, p > 0.15) and the time between implantation and first MEG measurement (r(11) = −0.12, p > 0.7) were all uncorrelated with the relative dipole displacement from T1 to T2.

**Figure 4:**
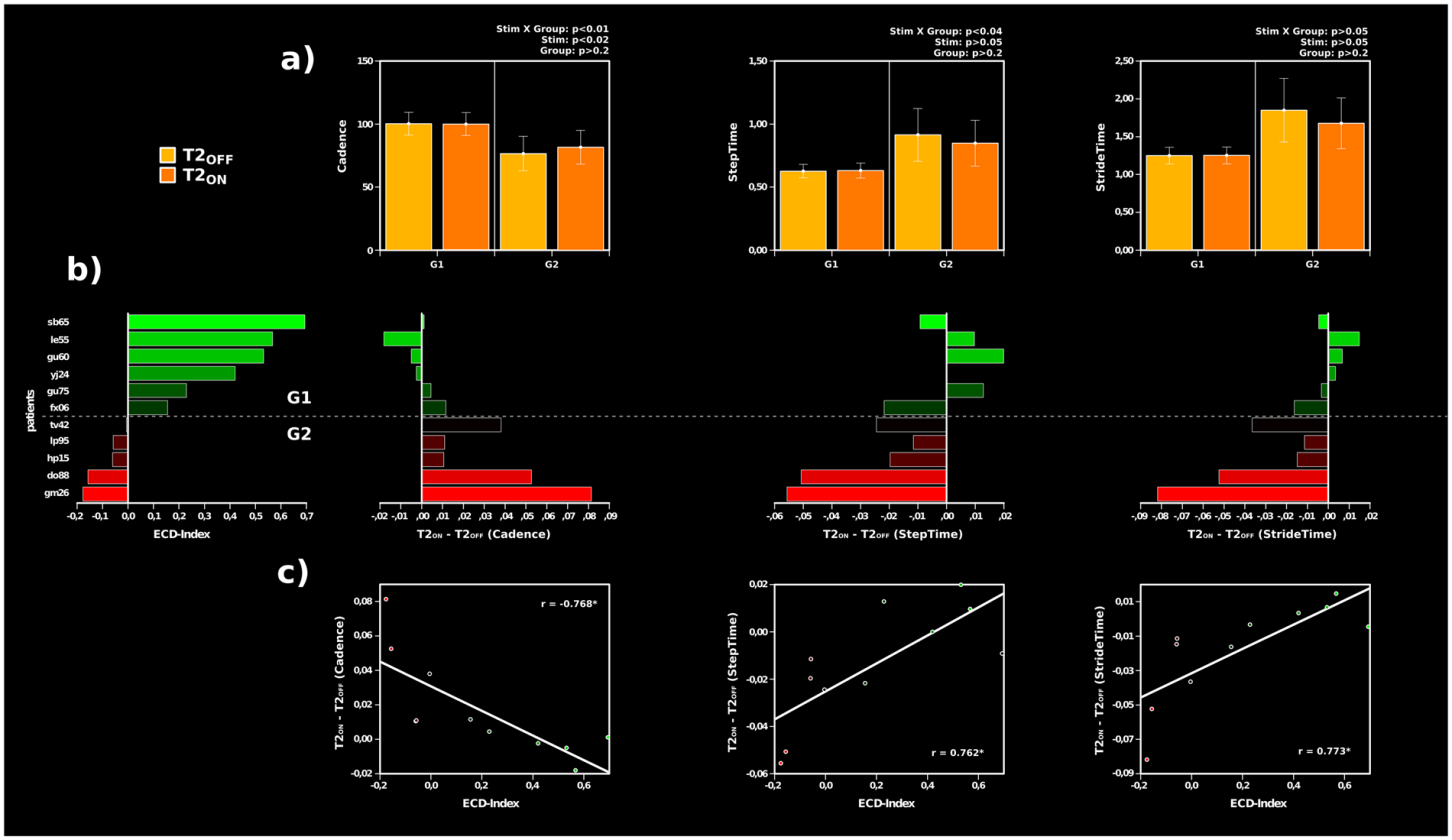
Gait and localization. Correlation between gait performance and laterality of cortical plasticity. (**a**) For G1 the gait performance did not decline, once the stimulator was turned off. However gait performance droped without active stimulation in G2. (**b**) This decline was highly correlated with a larger dipole displacement in the contralesional hemisphere. The vertical barplots show the ECD-Index and relative gait performance at T2 for each single patient. (**c**) The scatterplots display the relation between the ECD-Index and the change in gait performance at T2.

Subsequently, the ECD-Index was correlated with the relative change of gait performances over time (T1_OFF_ vs. T2_OFF_) and over stimulation at T2 (T2_OFF_ vs. T2_ON_) to determine the degree of which the relative plasticity of the sensorimotor cortices is related to the improvement of gait parameters by using the stimulator. Interestingly the general improvement of gait parameters over time was not related to the plasticity-index (cadence: r(11) = −0.16, p > 0.6; steptime: r(11) = 0.26, p > 0.4; stridetime: r(11) = 0.18, p > 0.5). However, the similarity of gait parameters at T2 in the case of active and inactive stimulation (T2_OFF_ vs. T2_ON_) was highly correlated with the ECD-Index (more cortical reorganization within the lesioned hemisphere) (cadence: r(11) = −0.77, p < 0.005; steptime: r(11) = 0.76, p < 0.006; stridetime: r(11) = 0.77, p < 0.005) (Fig. 4). To further analyze this very interesting effect, we subdivided the 11 patients into two groups, with one showing higher plasticity effects ipsi-lesional (positive ECD-values: G1) while the other included patients with more reorganization effects within the contra-lesional hemisphere (negative ECD-values: G2).

First, improvement in gait was assessed using two-factorial mixed model rANOVAs with the within factor time (T1_OFF_/T2_OFF_) and the between factor group. In line with the previous t-tests, subjects showed a general improvement in gait over time (cadence: F(1, 9) = 6.73, p < 0.029; steptime: F(1, 9) = 14.41, p < 0.004; stridetime: F(1, 9) = 12.72, p < 0.006). Additionally, as suggested by the correlation analysis with the ECD-Index there were no interaction-effects with the between-factor group (cadence: F(1, 9) = 0.36, p > 0.5; steptime: F(1, 9) = 1.08, p > 0.3; stridetime: F(1, 9) = 0.75, p > 0.4).

The same rANOVAs with the within factor stimulation (T2_OFF_/T2_ON_) and the between factor group revealed quite different results (Fig. 4). The main effect for stimulation was significant for cadence (F(1,9) = 9.30, p < 0.014) but failed to reach significance for steptime (F(1,9) = 4.46, p > 0.06) and stridetime (F(1,9) = 4.14, p > 0.07). Most interestingly the stimulation at T2 interacted with group for cadence (F(1,9) = 11.33, p < 0.008) and steptime (F(1,9) = 6.05, p < 0.036). This interaction effect stems from the improved gait parameter of cadence for T2_ON_ compared to T2_OFF_ in group 2 (t(10) = 3.93, p < 0.017) but not in group 1, for which the cadence does not differ between the stimulation ON and stimulation OFF conditions (t(10) = −0.26, p > 0.8).

## Discussion

The present study investigated the neural correlates of motor improvements associated with the usage of an implanted active prosthesis in patients with drop-foot following brain lesions. Typically, these patients have trouble to walk and fall down often due to the inability to lift the foot. The prosthesis consists of a switch that is usually placed in a sock under the heel, a central control unit and an implantable device that performs electrical stimulation over the peroneal nerve. In short, depending on the setup the switch triggers the electrical stimulation either when the heel is lifted or when it touches the ground and the foot is lifted due to the muscular contraction of the tibialis anterior and peroneal muscles. A common observation in patients is, that they constantly improve or adapt their gait to the action of the prosthesis in the first weeks following implantation. Changes in the activity of the tibialis anterior can be linked directly to efferent changes in the central nervous system, as electrophysiological signals of the somatosensory cortex and signals of the proximal muscle fibers are highly coherent during gait^33, 34^. This link is impaired in stroke patients^35^.

Here we employed magnetencephalography in conjunction with somatosensory stimulation of the hand in order to map the hand representation in the primary somatosensory cortex around the time of the implantation and about three to four months later, during which the patients improved their gait using the active prosthesis. Given that in most patients the foot representation in the somatosensory cortex was damaged, sensory evoked fields related to foot stimulation exhibited signal-to-noise-ratios that prevented any reasonable mapping of the foot representation within the somatosensory cortex. The idea was that plastic changes in the somatosensory representation of the foot due to walking with the active prosthesis would affect the representation of the hand and shift it towards ventral and lateral. All patients showed improved gait with the active prosthesis after three to four months. Patients exhibiting plastic changes predominantly within the lesioned hemisphere during that time exhibited gait improvements that did not decline when the prosthesis was inactive. Patients with predominantly contralesional plastic changes also improved; however their gait performance worsened when the prosthesis was turned off.

In the literature motor recovery processes after stroke are divided into an acute/sub-acute phase of about 6 months which is characterized by changes in the functional organization paralleled by clinical improvement and a chronic phase (not later than 12 months after the lesion) during which only little improvement of clinical measures occurs^36, 37^. Changes in cortical plasticity patterns during the sub-acute stage assessed with fMRI^29, 32, 38^ were shown to decrease considerably towards the onset of the chronic stage^39^. Importantly the current study draws a very different picture. Here, the lesion leading to the drop-foot occurred more than 2 years ago in all patients. The observed plastic changes argue strongly that the functional electric stimulation (FES) due to the active prosthesis triggers neural plasticity in chronic phase patients. The results confirm previous reports of therapeutic improvements of gait after FES-training in chronic stroke patients^4, 9, 17^ and provide strong evidence for cortical reorganization processes also in this phase that are linked to an improvement of clinical parameters.

We observed two types of functional reorganization in our patients. One group of patients showed changes in the functional representation of the hand in the sensorimotor cortex predominantly in the ipsilesional hemisphere. The representation moved towards ventral and lateral. In these patients the gait improvement persisted when the active prosthesis was inactive. After about 3 months of daily use of the active prosthesis most parameters like cadence or stride length were improved and importantly did not change much between the ON and the OFF condition. This clearly indicates that the usage of the prosthesis had a clear therapeutic effect^8^ in this patient group. The second group of patients showed changes in the functional representation of the hand predominantly in the contralesional hemisphere. In these patients there was also a considerable improvement of the gait parameters after about 3 months, however, when the active prosthesis was turned off the gait parameters immediately declined. This was indicated by clear differences in cadence between the ON and the OFF condition at the second measurement time point.

Previous studies employing electrophysiological measures reported large changes in the sensorimotor representations of stroke patients resulting in a hemispheric asymmetry during their acute and sub-acute stage. The asymmetry was mainly caused by displacements of the representation within the lesioned hemisphere^40, 41^. Interestingly, higher asymmetries during the first weeks after the stroke were related to better prospective motor-recovery rates^42, 43^. Functional imaging studies largely confirmed these results. A common measure to assess cortical hemispheric recruitment in stroke patients is the relative laterality of sensorimotor cortex (SMC) strength^30, 31, 44^. Longitudinal studies tracing patients during their acute stage consistently found an increase of laterality, thus a refocusing from bilateral SMC activity towards the ipsilesional SMC^29, 38, 45, 46^. Importantly, fully recovered patients showed a larger focusing on the ipsilesional side^47, 48^. This is fully in line with our findings in the patients who improved and maintained the level of improvement when the prosthesis was turned off. The change in cortical representation of the hand was towards ventral and lateral, which is also consistent with previous reports^48, 49^. It is assumed that after an initial recruitment of bilateral SMC shortly after the stroke to compensate for the lesion, a perilesional spread of the damaged representation into adjacent topographical maps occurs^50, 51^. This process of intrahemispheric reorganization is largely dependent on implicit, repetitive motor-learning^50, 52, 53^. It appears reasonable to assume that active prosthesis-assisted walking triggered such reorganization processes in this patient group.

The second group of patients did not maintain their level of behavioral improvement when the prosthesis was turned off. Plastic changes in the SMC of this group was much less lateralized and, if at all, more prominent in the contralesional hemisphere. This finding is also fully consistent with the literature. Functional imaging studies suggest that patients with persistent bilateral SMC recruitment^39^ seize to show large improvements in motor recovery^29, 32^. In these patients the initial bilateral activation patterns to compensate for the lost sensorimotor networks appear to solidify. Importantly, these patients also learn to use the active prosthesis but cannot restore gait-patterns without the active prosthesis.

This study exhibits the underlying neural mechanisms of improved motor functions after walking with an active prosthesis in upper motor lesion patients. It provides evidence that cortical reorganization processes can be reinitialized in chronic patients using such devices and potentially improve motor functionality. Even when device-assisted walking does not induce specific reorganization processes within the lesioned hemisphere, the gait is still improved with the active prosthesis. Future research in a larger group of patients is needed in order to find out how stable the behavioral improvement remains over time in patients when the active prosthesis is turned off. If the effect is stable, then gait analysis could be used to guide the choice of the active prosthesis. Unless there are other important medical reasons for an implantable device (such as for the patients in the current study) all patients could first receive a non-implantable active prosthesis. Patients who maintain gait improvements for a longer time after the device is turned off would probably not need an implantable device. Patients in whom the gait improvements rely on the activity of the device would benefit more from the implantable one.

### Data availability

The datasets generated and analyzed during the current study are available from the corresponding author on reasonable request.
